# Scaling for quantum tunneling current in nano- and subnano-scale plasmonic junctions

**DOI:** 10.1038/srep09826

**Published:** 2015-05-19

**Authors:** Peng Zhang

**Affiliations:** 1 Department of Nuclear Engineering and Radiological Sciences, University of Michigan, Ann Arbor, Michigan 48109-2104, USA

## Abstract

When two conductors are separated by a sufficiently thin insulator, electrical current can flow between them by quantum tunneling. This paper presents a self-consistent model of tunneling current in a nano- and subnano-meter metal-insulator-metal plasmonic junction, by including the effects of space charge and exchange correlation potential. It is found that the *J*-*V* curve of the junction may be divided into three regimes: direct tunneling, field emission, and space-charge-limited regime. In general, the space charge inside the insulator reduces current transfer across the junction, whereas the exchange-correlation potential promotes current transfer. It is shown that these effects may modify the current density by orders of magnitude from the widely used Simmons’ formula, which is only accurate for a limited parameter space (insulator thickness > 1 nm and barrier height > 3 eV) in the direct tunneling regime. The proposed self-consistent model may provide a more accurate evaluation of the tunneling current in the other regimes. The effects of anode emission and material properties (i.e. work function of the electrodes, electron affinity and permittivity of the insulator) are examined in detail in various regimes. Our simple model and the general scaling for tunneling current may provide insights to new regimes of quantum plasmonics.

Electron tunneling between plasmonic resonators is recently found to support quantum plasmon resonances^1^,^2^,^3^,^4^,^5^, which may introduce new regimes in nano-optoelectronics, nonlinear optics, and single-molecule sensing. Tunneling conductivity is also important in the recently proposed transition voltage spectroscopy (TVS)^6^,^7^,^8^, self-assembled monolayer (SAM)-based tunneling junctions^9^, resistive switching^10^, carbon nanotube (CNT) and graphene based electronics^11^,^12^,^13^. Tunneling effects between electrodes separated by thin insulating films have been studied extensively by Simmons^14^,^15^,^16^,^17^,^18^ in 1960s. Simmons’ formula^14^ have since been used as the basic scaling for evaluating tunneling current. The tunneling current in Al-Al_2_O_3_-Al structures has been experimentally studied and evaluated using Simmons’ theory^19^. Tunneling current of metal-oxide-semiconductor structures was also calculated using first-principle approaches^20^. An excellent review on the tunneling current in metal-insulator-metal structures is given in Ref^21^. However, Simmons’ formulas^14^ are derived by considering only the emission process from the electrodes, where the effects of image charge are considered, but the electron space charge potential and the electron exchange-correlation potential inside the insulator thin films are generally ignored. Thus, its accuracy in various regimes is largely unknown^9^,^22^. On the other hand, the effects of space charge in a vacuum nanogap have recently been studied extensively^4^,^23^,^24^, with extensions to short pulse^25^ and higher dimensions^26^,^27^. However, these studies assumed that current emission was only from the cathode (electrode with lower bias). The current emission from the anode (electrode with higher bias) (Fig. 1), which will be shown later (Fig. 2) that[Fig f3]sometimes can become comparable with the cathode current, was ignored^4^,^28^. Thus, there is still lack of a self-consistent model to systematically characterize the quantum tunneling current in a nano- and subnano-scale tunneling junction, including the effects of different insulating materials. This paper provides such a study, over a wide range of insulator film thickness, applied voltage, and material properties.[Fig f4]

It is found that Simmons’ formula is only accurate in a limited parameter space in the direct tunneling regime (Figs. 2, 3, 4, 5), when the insulating thin film is relatively thick (>1 nm) and the barrier height is relatively large (>3 eV). Its accuracy decreases when the effective barrier height decreases (Fig. 4), or when the permittivity of the insulator decreases (Fig. 5), where the self-consistent model would provide a more accurate prediction of the tunneling current. In the field emission regime and space-charge-limited regime, the self-consistent model may be used, as Simmons’ formula becomes fairly unreliable. The proposed model reveals the general scaling for quantum tunneling current and its dependence on the bias voltage, the dimension and material properties of the tunneling junction. It can be applied to broad areas involving tunneling junctions. As an example, its application in quantum plasmonics will be briefly addressed in the Discussion Section.

Note that although the present model is developed for DC condition, it is applicable to plasmonics of up to Near Infrared frequency. The underlying reason is that the transit time for electron tunneling through a barrier of nm-scale thickness is typically less than 1 fs^4^,^29^,^30^,^31^, which is much shorter than the period of the driving fields (e.g. 10 fs for 0.4 eV optical energy). This transit time is even shorter for insulator of sub-nm thickness. Thus, the electron would see an almost constant barrier during its transit time, and the DC model applies. Such treatments have been extensively applied in quantum plasmonic modeling^2^,^3^,^4^. The DC calculation would not be valid if the driving field frequency is so high (e.g. Visible light frequency or higher) that its period is comparable or less than the electron transit time.

## Results

### Self-consistent model for tunneling current

**Figure 1:**
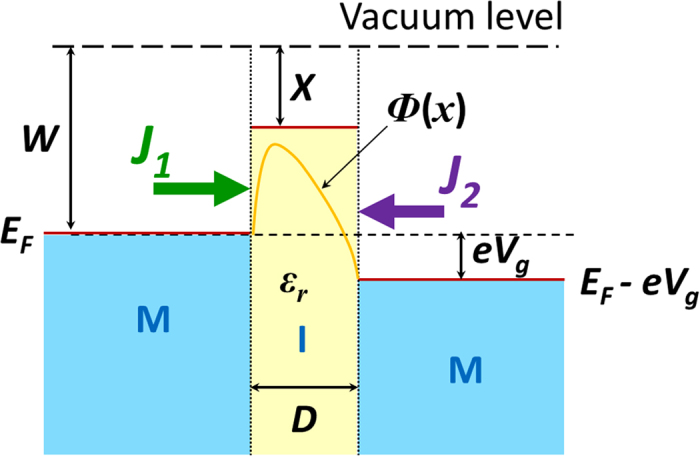
Metal-insulator-metal tunneling junction. The metal electrodes have Fermi level 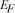 and work function 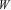. The insulator thin film has electron affinity 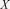, relative permittivity 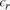, and thickness 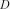. The applied voltage bias is 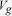, the effective potential between the electrodes is 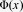. The current densities emitted from the cathode and the anode into the gap are 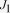 and 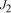, respectively.

Consider two metallic electrodes separated by a thin insulating film, as shown in Fig. 1. Since the insulating film is assumed to be sufficiently thin (in the subnano- and nano-meter scale), charge trapping may be ignored^17^,^32^. The electrons in the electrodes would see a potential barrier formed between the two electrodes,
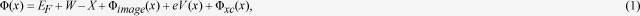
where 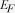 and 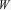 are the Fermi level and the work function of the metal electrodes respectively; 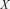 is electron affinity of the insulator; 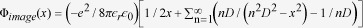 is the image charge potential energy including the effect of anode screening^4^,^14^,^33^, where 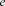 is the electron charge, 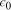 is the permittivity of free space, 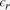 is the relative permittivity of the insulator, and 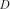 is the gap distance; 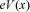 is the electric potential, which is the sum of the potential due to the external applied voltage 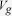 and the potential due to the electron space charge; and 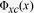 is the electron exchange-correlation potential, where the exchange potential is related to the Pauli Exclusion Principle, and the correlation potential denotes the quantum-mechanical part of the Coulomb interaction between electrons. The term 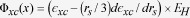 is calculated by Kohn-Sham local density approximation (LDA)^34^, where 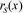 is the local Seitz radius 
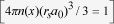
 in terms of the Bohr radius 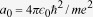 = 0.0529 nm, 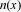 is the electron density, 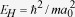 = 27.2 eV is the Hartree energy, 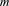 is the electron rest mass, 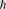 is the reduced Planch constant, and 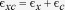 is the exchange-correlation energy^34^,^35^,^36^. Here, 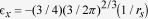, and 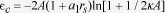 are the exchange energy^35^ and the correlation energy^34^ respectively, for a uniform electron gas of density 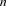 under the Kohn-Sham LDA assumption, where 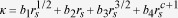, and 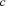, 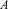, 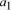, 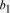, 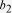, 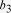, and 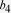 are parametrized constants obtained using the random phase approximation^34^.

Following Simmons^14^, the probability 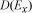 that an electron with longitudinal energy 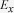 (normal to the surface) can penetrate the potential barrier of height Φ(*x*) is given by the WKBJ approximation^37^,
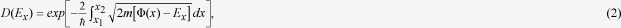
where 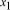 and 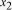 are the two roots of 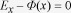. The current density tunneling through the barrier from electrode 1 to the right is calculated by^4^,^14^,^28^,^38^,^39^,
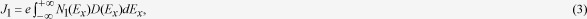

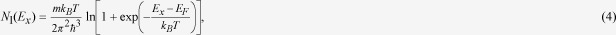
where 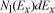 is the total number of electrons inside electrode 1 with longitudinal energy between 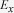 and 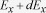 impinging on the surface of electrode 1 across a unit area per unit time, calculated by free-electron theory of metal^40^, with 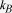 and 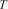 being the Boltzmann constant and the temperature, respectively.

Similarly, the current density tunneling through the barrier from electrode 2 to the left is^14^,^41^,
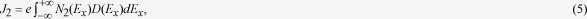

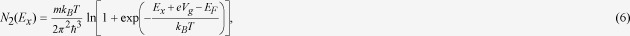
where 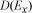 is given in eq. (2), and 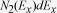 is the total number of electrons inside electrode 2 with longitudinal energy between 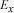 and 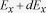 impinging on the surface of electrode 1 across a unit area per unit time, calculated by free-electron theory of metal^40^.

Inside the insulator between the two electrodes, 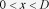, we use the mean-field theory^23^,^24^,^25^ to solve the electric potential 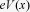 and the exchange-correlation potential 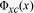, as appeared in eq. (1). Thus, we solve the coupled Shrodinger equation and the Poisson equation^23^,^24^,^25^,
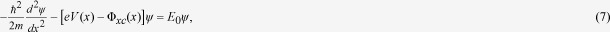

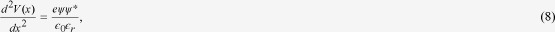
where 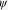 is the complex electron wave function, 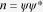 is the electron density, and 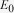 is the electron emission energy (with respect to the Fermi energy 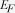). Note that 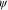 is the superposition of two streams of electrons, one travelling from electrode 1 to electrode 2, and the other from electrode 2 to electrode 1 (Fig. 1), both with emission energy of 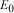. We assume 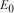 = 0 in the calculation^4^,^23^,^24^,^28^.

For a given gap bias voltage 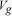, we have 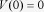, and 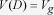. The boundary conditions on the wave function 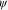 are derived from the conditions that 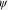 and 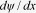 are continuous at 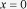, and 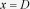. Charge conservation requires that the current density J_net_ = e(iħ/2m) (ψψ*′ − ψ*ψ ′) = J_1_ − J_2_ be constant for all 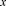, where a prime denotes a derivative with respect to 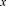, and 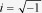.

It is convenient to introduce nondimensional quantities, 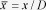, 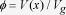, 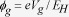, 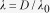, 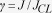, 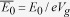, 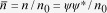, 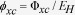, where 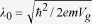, 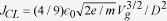 is the Child-Langmuir law^42^,^43^, 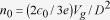, and 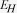 is the Hartree energy. The wave function may be expressed in the normalized form to read^23^, 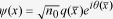, where 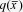 and 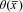 are respectively the nondimensional amplitude and phase, both assumed real. Thus, the coupled Shrodinger equation and the Poisson equation, eqs 7 and 8, are expressed in their normalized form as,
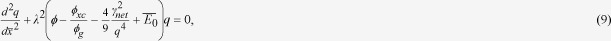

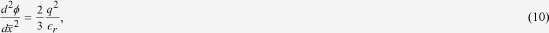
where 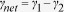 is the net normalized current density in the metal-insulator-metal (MIM) tunneling junction. The boundary conditions to eqs. (9) and (10) read,
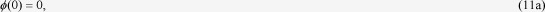

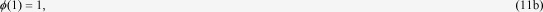

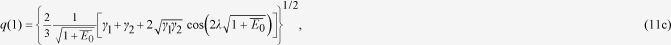

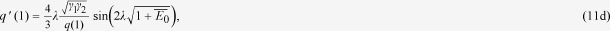
where eqs. 11c and 11d are derived by matching the wave function and its derivative at 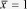. The normalized emission current density 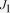 and 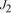 in eqs. (3) and (5) are,
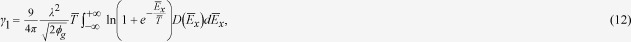

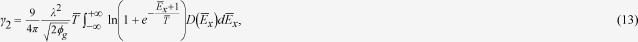
where 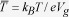, 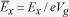. Note that the integrations in eqs. (12) and (13) are independent of the Fermi level 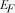.

By solving eqs. (9)-(13), 91, 93, 94, 95, 96, 100, 101 iteratively, we are able to self-consistently obtain the numerically converged results of the complete potential barrier profile of Φ(*x*) [eq (1)], the current density emitted from both electrodes 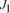, 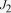, and therefore the net current density 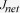, for any given materials of the electrodes (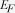, 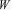), thin film insulator (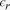, 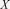), film thickness (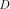), and external applied bias voltage (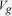). This is referred as the self-consistent method (SCM) thereafter.

### Main results

**Figure 2:**
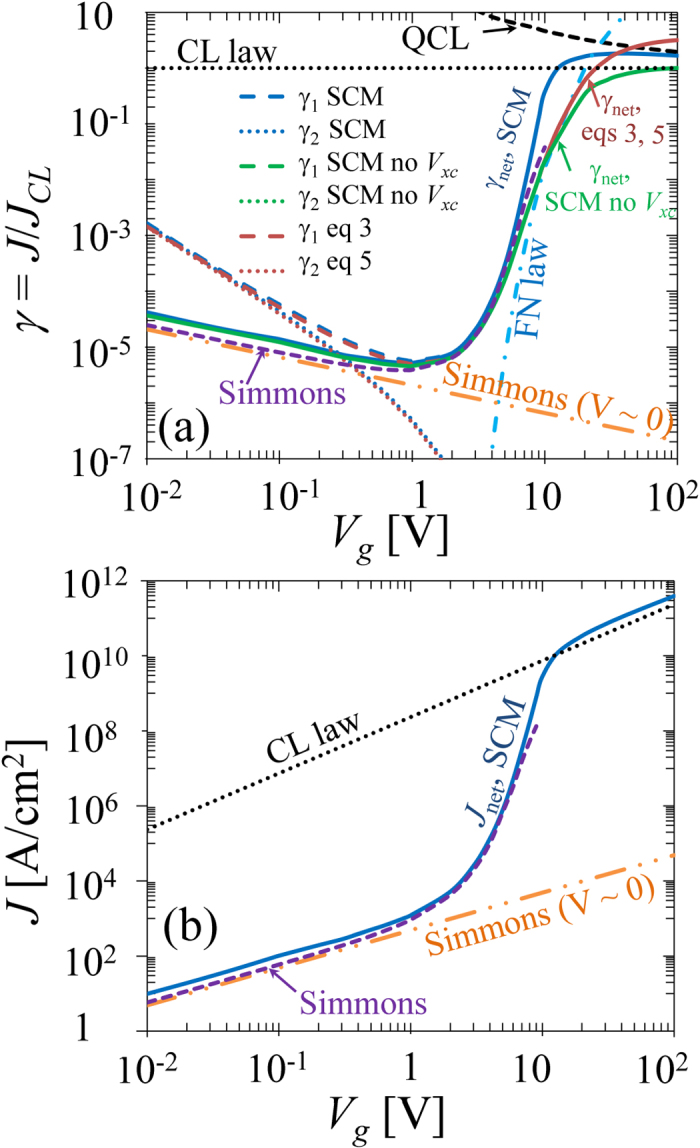
Current density as a function of applied gap voltage 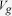, for two gold (Au) electrodes (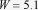 eV)^39^ separated by a vacuum gap (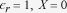 eV) of width 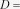 1 nm, at 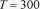 K, (**a**) in normalized form in terms of CL law, 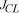, (**b**) in unit of A/cm^2^. The calculations in (**a**) are from three methods: direction integration of eqs 3 and 5 (or 12 and 13), SCM with no 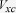 included, and full SCM with both space charge and 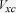 included. Simmons is for eq. 14, Simmons (V ~ 0) is for eq. 15.

Figure 2a shows the normalized current density 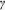 (in terms of CL law) as a function of applied gap voltage 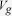, for two gold (Au) electrodes (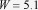 eV)^39^ separated by a 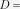 1 nm vacuum gap (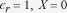 eV). The current density in A/cm^2^ is shown in Fig. 2b. The current densities are calculated from three methods: (1) direct integration using eqs 3 and 5, where space charge potential and exchange correlation potential 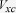 are not included in eq 1, (2) SCM without 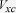, i.e. only space charge potential is included, and (3) complete SCM with both space charge potential and exchange correlation potential 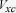 included. As shown in Fig. 2, the 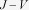 curves may be roughly divided into three regimes: direct tunneling regime (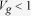 V), field emission regime (1 V 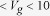 V), and space-charge-limited regime (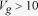 V).

In the direct tunneling regime, the tunneling current density from cathode 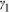 and that from anode 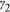 are comparable, where the latter was ignored in Refs. 4,25,28. The net current density, which is the difference between 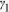 and 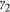, 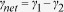, may therefore be orders of magnitude lower than both 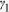 and 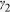. Thus, in the direct tunneling regime, both anode emission and cathode emission have to be considered to give an accurate evaluation in the tunneling current of the junction. The difference between 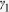
and 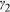 increases as 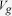 increases. The three methods mentioned above give almost identical results for the current densities 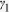, 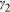, and 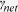 when 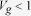 V, which implies that the space charge potential and exchange-correlation potential are not important in the direct tunneling regime, for the given Au-vacuum-Au junction with 1 nm gap spacing. The 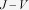 characteristic in the direct tunneling regime is linear, which indicates that the tunneling junction acts like an ohmic resister. The results are compared with the Simmons formula^14^,^19^,^21^ for general 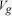,
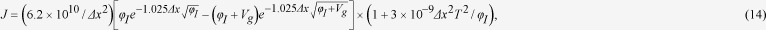
where 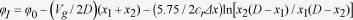, 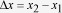, 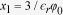, and 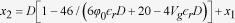 if 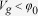, and 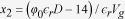 if 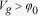, with 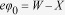. In the limit of small bias voltage, 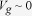, Simmons derived a simpler formula^14^,^21^,
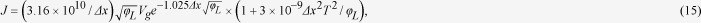
where 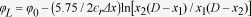, 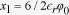, and 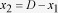. The last term in eqs 14 and 15 shows the temperature dependence of the tunneling current. In eqs 14 and 15, 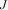 is in A/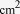, 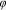 in V, 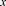 in Å, and 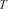 is in K. Equation 15 show clearly a linear 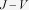 dependence, which is also plotted in Fig. 2. Despite a slight down shift (<30%) in results of the Simmons formulas (which can be easily adjusted, e.g. by replacing the constants with larger values), eqs. (14) and (15) give a fairly good estimation in the 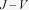 behavior of the given Au-Vacuum-Au structure when the applied bias 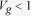 V. It has been checked that the 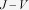 curves for the Au-Vacuum-Au structure in Fig. 2 is very insensitive to temperature: only with an increase of < 2% from 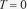 K to 600 K, which is consistent with the relative small *T* dependence in eqs. 14 and 15. Physically, this is because the apparent barrier height of the Au-Vacuum-Au structure (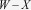 = 5.1 eV) is much higher than the width change of the Fermi function (~0.5 eV), so that the majority electrons would still see an almost unchanged tunneling barrier for 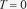 K to 600 K. The temperature dependence would become important for junctions with small barrier heights (e.g. 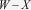 ~1 eV).

In the regime of 1 V 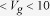 V, the tunneling current from anode 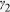 is much smaller compared to the cathode current 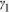. By applying an appreciable bias voltage 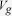, the effective barrier height for the cathode is reduced, indicating an increase of current 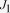 or 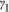 with 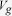. However, due to the down shift of the “effective” Fermi level (Fig. 1), the effective barrier height seen by electrons in the anode is increased, leading to a dramatic drop of current 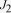 or 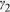 with 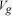. The tunneling behavior of the junction resembles field emission, thus we denote this regime the field emission regime. Field emission is most widely modeled by Fowler-Nordheim (FN) law^38^,^44^,^45^, 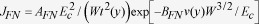, where 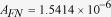
AeVV^−2^ and 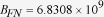 eV^−3/2^Vm^−1^, 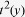 and 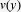 are Nordheim parameters with 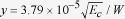, and 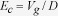 is the applied electric field. FN law is derived by assuming no anode screening. As shown in Figure 2, although the net current density 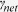 is approaching the FN law as 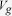 increases, in general FN law is not sufficiently accurate to model the tunneling current in such a nano-scale junction^28^. In this regime, Simmons formula, eq. (14), gives a more accurate fit to the self-consistent SCM result. Note the breakdown of eq. (14) around 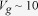 V, where the effective barrier height is depressed by 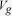 below the Fermi level of the cathode. When 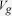 is approaching 10 V, the current from direct integration (eqs. (3) and (5)) is closely fitted by Simmons formula, eq. (14). The current calculated from SCM by including only the space charge effect is slightly reduced. However, when both exchange-correlation 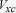 and space charge effects are included in the SCM, the resulting current is enhanced by one order of magnitude, indicating the profound effect of exchange-correlation energy in the field emission regime.

In the space-charge-limited regime of 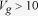 V, the direct integration method, which ignores both the space charge effect and the exchange-correlation effect, cannot provide a reliable estimate of the current. When only the space charge potential is included in the SCM calculation, the resulting current is reduced and is approaching classical Child-Langmuir (CL) law, 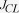. However, when exchange-correlation potential is also included in the SCM calculation, the emitted current is enhanced in general. When 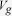 reaches 100 V, the cathode current 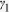
(and therefore the net current 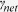) approaches the quantum CL law (QCL)^23^,^24^, which gives the maximum current density that can be transported across a vacuum nano-gap for a given 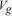 and 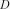, with quantum corrections.

**Figure 3 f3:**
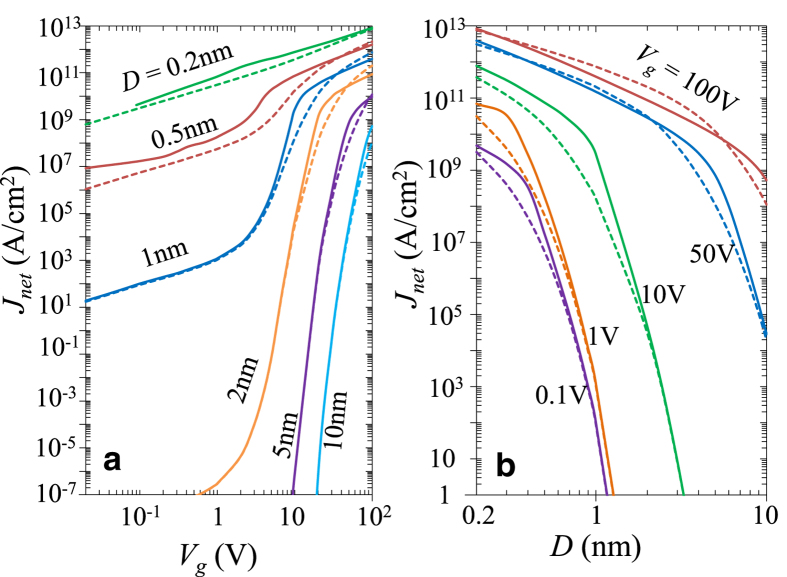
The effects of gap width 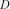
on the 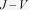 characteristics of the Au-Vacuum-Au junction. (**a**) 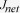 as a function of 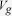, for various 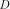, (**b**) 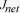 as a function of 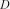, for various 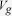. Solid lines are from SCM, dashed lines are from direct integration of eqs. (3) and (5). For gold (Au) electrodes^39^, 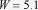 eV, for vacuum gap, 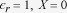 eV.

Figure 3a shows the net current density 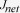 as a function of 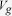, for various gap width 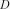 for the Au-Vacuum-Au tunneling junction. Similar to Figure 2, the 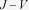 curve may be roughly divided into three regimes: direct tunneling regime, field emission regime, and space-charge-limited regime. As gap width 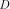 decreases, the voltage range for both the direct tunneling regime and the space-charge-limited regime expands towards the field emission regime, whose voltage range decreases with 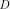. In the direct tunneling regime, when 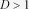 nm, the direct integration method and the SCM give almost identical results, where the Simmons formula (eq. (14)), which fits the direct integration well, is a very good approximation. However, when the gap width is in the sub nanometer range, 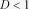 nm, the direct integration method (and therefore Simmons formula) underestimates the net current, thus the SCM including the effects of both space charge and exchange-correlation needs to be used to give more accurate calculation. In general, direct integration method would not be accurate in the field emission regime and space-charge-limited regime, where the SCM has to be applied. In the space-charge-limited regime, 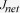 approaches QCL limit as 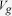 increases.

Figure 3b shows the net current density 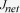 as a function of insulator thin film thickness 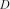, for various 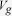 for the Au-Vacuum-Au tunneling junction. It is important to see that the tunneling current, therefore the tunneling conductivity, is extremely sensitive to the thickness of the insulating thin film in MIM tunnel junctions. It is clear that for the limited parameter space, e.g. 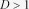 nm and 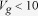 V, the direct integration calculation is accurate. Note that the values of gap voltage 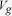 and gap spacing 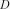 in Fig. 3 are within the typical range of quantum plasmonic applications^3^,^4^,^39^.

**Figure 4 f4:**
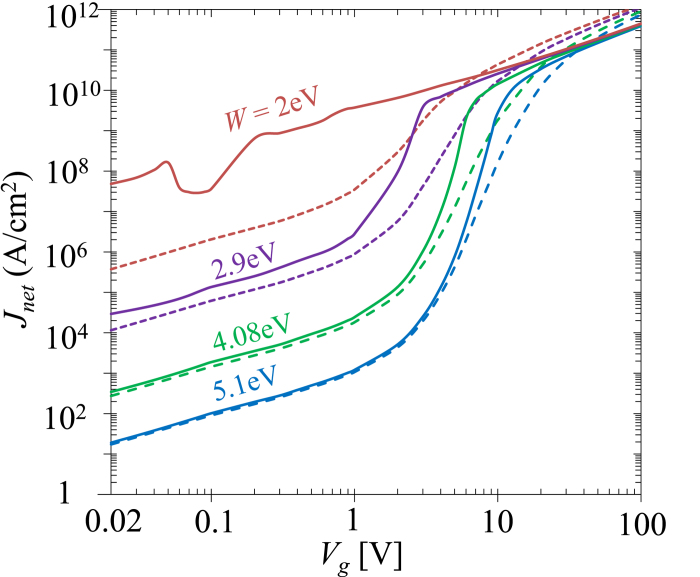
The effects of work function 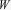 on the 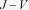 characteristics of a MIM junction with 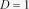 nm Vacuum gap. Top to bottom, 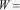 2 eV (Cs)^49^, 2.9 eV (Ca)^49^, 4.08 eV (Al)^50^, 5.1 eV (Au)^39^. For Vacuum gap, 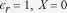 eV. Solid lines are from SCM, dashed lines are from direct integration of eqs. (3) and (5).

The 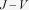 characteristics of a MIM junction (Figure 1) is very sensitive to its apparent barrier height, 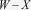. Figure 4 shows 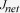 as a function of 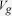 for MIM junctions formed by various metal electrodes separated by a 1 nm wide vacuum gap. When the work function of the electrodes increases from 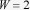 eV (Cs) to 5.1 eV (Au), 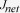 in the direct tunneling regime (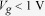) decreases by 6 orders of magnitude for a given bias. Simmons formula (eq. (14)) and the direct integration method are only accurate when 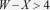 eV for a junction with vacuum gap 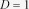 nm. When 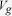 approaches 100 V, the current density 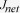 converges to the same asymptotic value of QCL, since the space-charge-limited current density depends only on 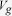 and 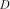, but not on 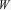. The effect of the electron affinity 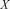 of the insulating thin film (Fig. 1) on 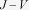
would be similar, that is, increasing 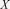 would be equivalent to decreasing 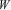, provided the relative permittivity 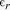 of the insulator is unchanged.

It is interesting to note the nonmonotonic behavior of some curves in Fig. 3a (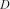 = 0.5 nm when 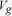
* < *1 V) and Fig. 4 (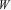 = 2 eV when 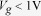). This is due to the profound effects of the nonlinear exchange-correlation potential, where the normalized insulating gap space 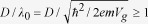, and the normalized gap voltage 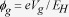 << 1 so that the space charge potential is not important compared to the exchange-correlation potential^24^,^25^,^28^,^36^,^46^.

**Figure 5:**
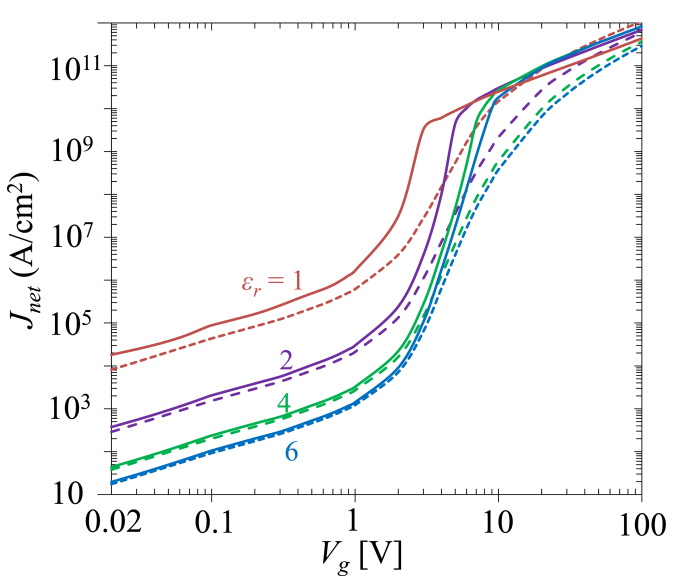
The effects of the relative permittivity 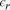 of insulating thin film on the 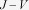 characteristics of a MIM junction with insulator thin film thickness 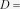 1 nm, for fixed apparent barrier height of 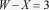 eV. Solid lines are from SCM, dashed lines are from direct integration of eqs. (3) and (5).

The effect of relative permittivity 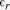 of the insulating thin film is shown in Fig. 5. In the direct tunneling regime (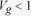 V), 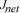 decreases as 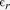 increases for a given 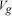. This is due to the fact that the image charge potential 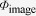 decreases as 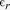 increases, as seen from the second line after eq. (1). Thus, the overall potential barrier will increase, leading to smaller tunneling current. In contrast, in the space-charge-limited regime (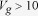 V), 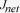 calculated by SCM (solid lines) increases with 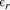, as clearly seen from Figure 5. This is because a larger 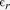 reduces the effect of space charge, as seen from eq. (8) or (10), thus resulting in a larger SCL current. Note that 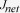 calculated by direct integration (dashed lines) shows very different trends from that of SCM, indicating the dominant effects of space charge, which have to be included to give reliable predictions in the space-charge-limited regime. Thus, Simmons formula and the direct integration method are only accurate in the direct tunneling regime, when 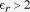 for junctions with 1 nm thickness and 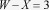 eV. It is important to note that if 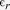 is temperature dependent, the 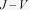 behavior would also be temperature dependent^14^, even for tunneling junctions with relative big barrier height.

## Discussion

Recently, the quantum-corrected model (QCM)^2^,^4^ has been introduced to study charge transfer plasmon (CTP)^47^,^48^ due to quantum tunneling, by accounting for the tunneling current across the gap via the insertion of an effective conductive medium in the gap. With the classical description, the permittivity 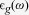 of the effective medium is related to its DC conductivity 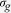 as 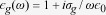, where 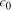 is the free space permittivity, and 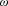 is the oscillating frequency. In the Drude model, the dielectric response of the effective conducting medium in the gap is characterized by 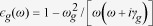, where 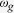 is the plasmon frequency (typically set to the bulk plasma frequency of the surrounding resonators), and 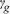 is the tunneling damping parameter, which can thus be calculated as 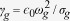, under the assumption that *γ*_*g*_>>*ω*. The optical responses and the induced local fields of the quantum plasmon system are then obtained by standard classical approaches solving Maxwell’s equations^2^,^47^. The validity of the calculation is crucially dependent on the two key parameters 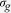 and 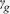, which describe the quantum tunneling resistance introduced by the presence of the gap.

**Figure 6:**
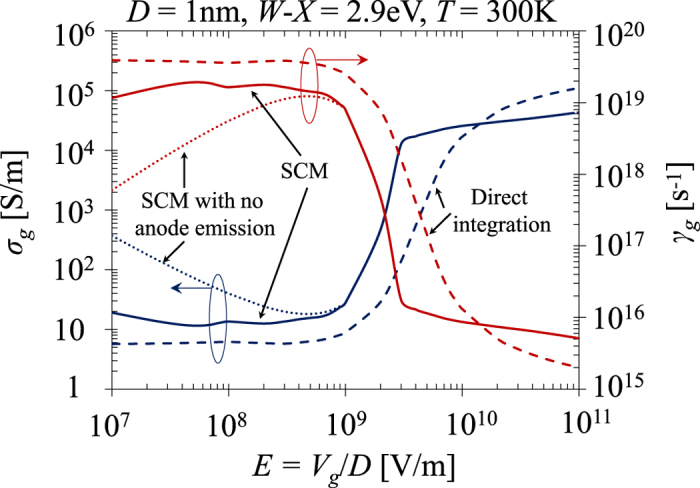
Quantum tunneling gap DC conductivity 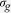 and the tunneling damping parameter 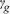 for a MIM plasmonic tunneling junction, as a function of applied electric field 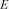, with a vacuum gap of 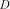 = 1 nm, 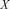 = 0, and work function of the electrodes 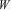 = 2.9 eV at 300 K. The plasmon frequency is assumed to be 
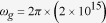
rad/s for the calculation of 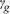. The solid lines are for SCM, dashed lines for direct integration of eqs 3 and 5, and dotted lines for SCM with anode emission being switched off (i.e. set 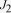 = 0 in eq 5 and 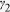 = 0 in eq 13).

As an example, in Fig. 6, 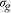 and 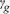 obtained from the proposed self-consistent model (SCM) are compared to those by direction integration (eqs. 3 and 5), and by the SCM but switching off the emission from anode (similar to Refs.^4^,^28^), for a tunneling junction with 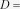 1 nm vacuum gap, and electrode work function 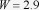 eV. For simplicity, we estimate the DC quantum gap conductivity as 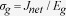, where 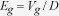 is the applied electric field across the tunneling gap. In direct tunneling regime (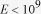 V/m) and the field emission regime (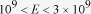 V/m), direct integration method (or Simmons formula) underestimates the gap conductivity and overestimates the tunneling damping. In the space-charge-limited regime (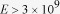 V/m), direct integration method is generally not reliable. Ignoring the current emission form anode (i.e. set 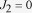 in eq 5) would result in a much higher 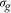 and much lower 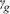 in the direct tunneling regime. The relative large damping 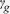 calculated from SCM in the direct tunneling regime suggests that CTP via quantum tunneling in this regime would be very difficult to observe experimentally. Instead, for a given junction, by simply increasing the driving field 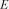 to reach the field emission or space-charge-limited regime, the damping 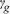 can be significantly reduced so that the experimental realization of CTP via tunneling could be relatively easier.

In summary, we have developed a self-consistent model to characterize the tunneling current of nano- and subnano-scale plasmonic junctions, by taking into account of the effects of both space charge and exchange-correlation potential. The effects of material properties, including the work function of the electrodes 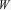, the permittivity 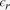 and the electron affinity 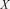 of the insulator, are examined in detail. In general, the 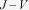 curves may be divided into three regimes: direct tunneling regime, field emission regime, and space-charge-limited regime. It is found that Simmons formula (eqs. (14) and (15)) are good approximations of the tunneling current for a limited parameter space in the direct tunneling regime only. Their accuracy decreases when the effective barrier height decreases, i.e. 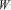 decreases or 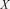 increases, or when the permittivity of the insulator 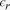 decreases. They become unreliable when the insulator thickness is in the sub-nanometer scale, 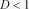 nm, where the self-consistent model would give a more accurate evaluation.

In this formulation, we have made the following widely used assumptions: 1) the electron transmission probability during the emission process is approximated by the WKBJ solution, where the metal electrodes are based on the free electron gas model; 2) the surfaces of the electrodes are flat and the problem is assumed one-dimensional; 3) the image potential is approximated by the classical image charge methods. The effects of electrodes geometry, nature of the ion lattice of the electrodes, possible charge trapping inside the insulator film, frequency dependence, and dissimilar electrodes will be subjects of future studies.

## Methods

N. A.

## Author Contributions

P. Z. conceived the idea, formulated the theory, performed the numerical calculations, and wrote the manuscript.

## Additional Information

**How to cite this article**: Zhang, P. Scaling for quantum tunneling current in nano- and subnano-scale plasmonic junctions. *Sci. Rep.* doi: **5**, 9826; 10.1038/srep09826 (2015).

## References

[b1] Savage K. J. *et al.* Revealing the quantum regime in tunnelling plasmonics. Nature 491, 574–577 (2012). 23135399 10.1038/nature11653

[b2] Esteban R., Borisov A. G., Nordlander P. & Aizpurua J. Bridging quantum and classical plasmonics with a quantum-corrected model. Nat. Commun. 3, 825 (2012). 22569369 10.1038/ncomms1806

[b3] Tan S. F. *et al.* Quantum Plasmon Resonances Controlled by Molecular Tunnel Junctions. Science 343, 1496–1499 (2014). 24675958 10.1126/science.1248797

[b4] Wu L. *et al.* Fowler-Nordheim tunneling induced charge transfer plasmons between nearly touching nanoparticles. ACS Nano 7, 707–716 (2013). 23215253 10.1021/nn304970v

[b5] Tame M. S. *et al.* Quantum plasmonics. Nat. Phys. 9, 329–340 (2013).

[b6] Huisman E. H., Guédon C. M., van Wees B. J. & van der Molen S. J. Interpretation of Transition Voltage Spectroscopy. Nano Lett. 9, 3909–3913 (2009). 19685928 10.1021/nl9021094

[b7] Trouwborst M. L. *et al.* Transition Voltage Spectroscopy and the Nature of Vacuum Tunneling. Nano Lett. 11, 614–617 (2011). 21214259 10.1021/nl103699t

[b8] Sotthewes K., Hellenthal C., Kumar A. & Zandvliet H. J. W. Transition voltage spectroscopy of scanning tunneling microscopy vacuum junctions. RSC Adv. 4, 32438–32442 (2014).

[b9] Nijhuis C. A., Reus W. F., Barber J. R. & Whitesides G. M. Comparison of SAM-Based Junctions with Ga2O3/EGaIn Top Electrodes to Other Large-Area Tunneling Junctions. J. Phys. Chem. C 116, 14139–14150 (2012).

[b10] Ziegler M., Harnack O. & Kohlstedt H. Resistive switching in lateral junctions with nanometer separated electrodes. Solid-State Electron . 92, 24–27 (2014).

[b11] Li C., Thostenson E. T. & Chou T.-W. Dominant role of tunneling resistance in the electrical conductivity of carbon nanotube–based composites. Appl. Phys. Lett. 91, 223114 (2007).

[b12] Bao W. S., Meguid S. A., Zhu Z. H. & Weng G. J. Tunneling resistance and its effect on the electrical conductivity of carbon nanotube nanocomposites. J. Appl. Phys. 111, 093726 (2012).

[b13] Sensale-Rodriguez B. Graphene-insulator-graphene active plasmonic terahertz devices. Appl. Phys. Lett. 103, 123109 (2013).

[b14] Simmons J. G. Generalized Formula for the Electric Tunnel Effect between Similar Electrodes Separated by a Thin Insulating Film. J. Appl. Phys. 34, 1793–1803 (1963).

[b15] Simmons J. G. Electric Tunnel Effect between Dissimilar Electrodes Separated by a Thin Insulating Film. J. Appl. Phys. 34, 2581–2590 (1963).

[b16] Simmons J. G. Potential Barriers and Emission‐Limited Current Flow Between Closely Spaced Parallel Metal Electrodes. J. Appl. Phys. 35, 2472–2481 (1964).

[b17] Frank R. I. & Simmons J. G. Space-Charge Effects on Emission-Limited Current Flow in Insulators. J. Appl. Phys. 38, 832–840 (1967).

[b18] Simmons J. G. Conduction in thin dielectric films. J. Phys. Appl. Phys. 4, 613 (1971).

[b19] Das V. D. & Jagadeesh M. S. Tunneling in Al-Al2O3-Al MIM structures. Phys. Status Solidi A 66, 327–333 (1981).

[b20] Zhang X.-G., Lu Z.-Y. & Pantelides S. T. First-principles theory of tunneling currents in metal-oxide-semiconductor structures. Appl. Phys. Lett. 89, 032112 (2006).

[b21] Kao K. C. Dielectric Phenomena in Solids . Academic Press 2004) p364

[b22] Joachim C. & Ratner M. A. Molecular electronics: Some views on transport junctions and beyond. Proc. Natl. Acad. Sci. U. S. A. 102, 8801–8808 (2005). 15956192 10.1073/pnas.0500075102 PMC1157019

[b23] Lau Y. Y., Chernin D., Colombant D. G. & Ho P.-T. Quantum extension of Child-Langmuir law. Phys. Rev. Lett. 66, 1446–1449 (1991). 10043211 10.1103/PhysRevLett.66.1446

[b24] Ang L. K., Kwan T. J. T. & Lau Y. Y. New Scaling of Child-Langmuir Law in the Quantum Regime. Phys. Rev. Lett. 91, 208303 (2003). 14683407 10.1103/PhysRevLett.91.208303

[b25] Ang L. K. & Zhang P. Ultrashort-pulse child-langmuir law in the quantum and relativistic regimes. Phys. Rev. Lett. 98, 164802 (2007). 17501425 10.1103/PhysRevLett.98.164802

[b26] Koh W. S., Ang L. K. & Kwan T. J. T. Three-dimensional Child–Langmuir law for uniform hot electron emission. Phys. Plasmas 1994-Present 12, 053107 (2005).

[b27] Zhu Y. B., Zhang P., Valfells A., Ang L. K. & Lau Y. Y. Novel Scaling Laws for the Langmuir-Blodgett Solutions in Cylindrical and Spherical Diodes. Phys. Rev. Lett. 110, 265007 (2013). 23848888 10.1103/PhysRevLett.110.265007

[b28] Koh W. S. & Ang L. K. Quantum model of space–charge-limited field emission in a nanogap. Nanotechnology 19, 235402 (2008). 21825791 10.1088/0957-4484/19/23/235402

[b29] Uiberacker M. *et al.* Attosecond real-time observation of electron tunnelling in atoms. Nature 446, 627–632 (2007). 17410167 10.1038/nature05648

[b30] Nimtz G. Tunneling Confronts Special Relativity. Found. Phys. 41, 1193–1199 (2011).

[b31] Thornber K. K., McGill T. C. & Mead C. A. The Tunneling Time of an Electron. J. Appl. Phys. 38, 2384–2385 (1967).

[b32] Rose A. Space-Charge-Limited Currents in Solids. Phys. Rev. 97, 1538–1544 (1955).

[b33] Smythe W. R. Static and dynamic electricity . McGraw-Hill 1950).

[b34] Perdew J. P. & Wang Y. Accurate and simple analytic representation of the electron-gas correlation energy. Phys. Rev. B 45, 13244–13249 (1992). 10.1103/physrevb.45.13244 10001404

[b35] Dirac P. a. M. Note on Exchange Phenomena in the Thomas Atom. Math. Proc. Camb. Philos. Soc. 26, 376–385 (1930).

[b36] Kohn W. & Sham L. J. Self-Consistent Equations Including Exchange and Correlation Effects. Phys. Rev. 140, A1133–A1138 (1965).

[b37] Bohm D. Quantum Theory . Courier Dover Publications 1951).

[b38] Jensen K. L. & Cahay M. General thermal-field emission equation. Appl. Phys. Lett. 88, 154105 (2006).

[b39] Haus J. W., de Ceglia D., Vincenti M. A. & Scalora M. Quantum conductivity for metal–insulator–metal nanostructures. J. Opt. Soc. Am. B 31, 259–269 (2014).

[b40] Omar M. A. Elementary Solid State Physics: Principles and Applications . Addison-Wesley 1994).

[b41] Cahay M., McLennan M., Datta S. & Lundstrom M. S. Importance of space‐charge effects in resonant tunneling devices. Appl. Phys. Lett. 50, 612–614 (1987).

[b42] Child C. D. Discharge From Hot CaO. Phys. Rev. Ser. I 32, 492–511 (1911).

[b43] Langmuir I. The Effect of Space Charge and Residual Gases on Thermionic Currents in High Vacuum. Phys. Rev. 2, 450–486 (1913).

[b44] Fowler R. H. & Nordheim L. Electron Emission in Intense Electric Fields. Proc. R. Soc. Lond. Ser. A 119, 173–181 (1928).

[b45] Murphy E. L. & Good R. H. Thermionic Emission, Field Emission, and the Transition Region. Phys. Rev. 102, 1464–1473 (1956).

[b46] Ang L. K., Lau Y. Y. & Kwan T. J. T. Simple derivation of quantum scaling in Child-Langmuir law. IEEE Trans. Plasma Sci. 32, 410–412 (2004).

[b47] Pérez-González O. *et al.* Optical Spectroscopy of Conductive Junctions in Plasmonic Cavities. Nano Lett. 10, 3090–3095 (2010). 20698622 10.1021/nl1017173

[b48] Zuloaga J., Prodan E. & Nordlander P. Quantum Description of the Plasmon Resonances of a Nanoparticle Dimer. Nano Lett. 9, 887–891 (2009). 19159319 10.1021/nl803811g

[b49] Haynes W. M. CRC Handbook of Chemistry and Physics , *94th Edition*. CRC Press 2013).

[b50] Tipler P. A. & Llewellyn R. Modern Physics . W. H. Freeman 2007).

